# The association of telomere length with substance use disorders: systematic review and meta-analysis protocol

**DOI:** 10.1186/s13643-019-1199-x

**Published:** 2019-12-01

**Authors:** Fernando Navarro-Mateu, María Rubio-Aparicio, Pedro Cayuela, Francisco-Javier Álvarez, Agustín Roca-Vega, María Dolores Chirlaque, María Luisa Cayuela, Mathilde Husky, Salvador Martínez, Julio Sánchez-Meca

**Affiliations:** 1 Unidad de Docencia, Investigación y Formación en Salud Mental (UDIF-SM), Servicio Murciano de Salud, c/ Lorca, n° 58, 30120 Murcia, Spain; 2 CIBER de Epidemiología y Salud Pública (CIBERESP), Madrid, Spain; 3 IMIB-Arrixaca, Murcia, Spain; 4 Departamento de Psicología Básica y Metodología, University of Murcia, Murcia, Spain; 5 Departamento Psicología de la Salud, University of Alicante, Alicante, Spain; 6 Escuela Universitaria de Enfermería de Cartagena, University of Murcia, Murcia, Spain; 7 Biblioteca Virtual MurciaSalud, Centro Tecnológico de Información y Documentación Sanitaria, Servicio Murciano de Salud, Murcia, Spain; 8 Servicio de Epidemiología, Consejería de Salud, Murcia, Spain; 9 Departamento de Ciencias Sociosanitarias, University of Murcia, Murcia, Spain; 10 Grupo Telomerasa, Cáncer y Envejecimiento, Hospital Clínico Universitario Virgen de la Arrixaca, Murcia, Spain; 11 Laboratoire de Psychologie EA4139, Université de Bordeaux, Bordeaux, France; 12 Instituto de Neurociencias, UMH-CSIC, Alicante, Spain

**Keywords:** Telomere length, Substance use disorders, Meta-analysis, Systematic review

## Abstract

**Background:**

The present protocol was designed for a systematic review and meta-analysis aimed at determining the association of telomere length with substance use disorders with the exclusion of nicotine addiction, and to identify potential moderators of the effect of telomere length. Such methodological information may provide guidance to improve the quality of future research on this important topic.

**Methods:**

Potential studies will be identified through electronic databases (PubMed/MEDLINE, EMBASE, PsycINFO, and Web of Science) up from inception onwards. The inclusion criteria will include published or unpublished observational studies (cohort, case–control, and cross-sectional studies) reporting telomere length in adult patients with substance use disorder compared with a control group. Non-human studies or other study designs such as reviews, case-only, family-based, and/or population studies with only healthy participants will be excluded, as well as those focused solely on nicotine addiction. The main outcome will be telomere length in adults with substance use disorder (primary) and, specifically, in those with alcohol use disorder (secondary). Two investigators will independently evaluate the preselected studies for possible inclusion and will extract data following a standardized protocol. Disagreements will be resolved by consensus. The risk of bias of all included studies will be assessed using the Newcastle–Ottawa Quality Assessment Scale for non-randomized studies. Data will be converted into standardized mean differences as effect size index, and random-effects models will be used for the meta-analysis. Cochran’s *Q* statistic, *I*^2^ index, and visual inspection of the forest plot will be used to verify study heterogeneity. Subgroup analyses and meta-regressions will be conducted to ascertain heterogeneity. Several sensitivity analyses will be conducted to address the influence of potential confounding factors. Publication bias will be examined using the “funnel plot” method with Duval and Tweedie’s trim-and-fill method and Egger test.

**Discussion:**

This systematic review will assess the association of telomere length with substance use disorders aside from nicotine addiction.

**Systematic review registration:**

PROSPERO registration number CRD42019119785

## Background

Human telomeres are repetitive noncoding DNA protein structures consisting of nucleotide sequences of tandem TTAGGG repeats at the end of chromosomes and associated protective proteins essential for maintaining genome stability [1, 2]. Telomere length is the result of the balance between telomere repeat additions and losses and is known to become shorter as a function of increased turnover and chronological age [3]. When telomere length becomes critically short, cell proliferation decreases and cell senescence ensues which has led telomere length to be considered as a marker of cellular aging [4–6]. Importantly, telomere length has been shown to be affected by genetic, epigenetic, and other non-genetic factors including exposure to stress and to socioeconomic or lifestyle-related factors [2, 7].

During the past decade, telomere attrition has been associated with increased morbidity and mortality of different age-related diseases, such as metabolic syndromes [8], diabetes mellitus [9], hypertension [10], increased risk of coronary heart disease [11, 12], and Alzheimer’s disease [13]. Short telomeres have also been associated with increased all-cause mortality risk in the general population [14]. Lastly, healthy lifestyles, including proper nutrition and higher levels of physical exercise, have been proposed to have protective effects on telomere length [15, 16].

Recently, there has been growing interest regarding the association of telomere length with known risk factors for mental illness including exposure to perceived stress [17], chronic social stress [18], and childhood adversities [19]. The association with mental disorders has also received particular attention [20, 21]. In recent meta-analyses, telomere length has been shown to be associated with depression [22, 23], bipolar disorder [24], post-traumatic stress disorder [19], anxiety [25], Alzheimer’s disease [13], schizophrenia [26, 27], and other psychiatric disorders (with the specific exclusion of substance use disorders (SUD)) [28]. The interest in studying telomere length lies not only in its potential as a biomarker for premature mortality or quality of life [20], but also in personalized medicine with implications for prognosis, treatment selection, and treatment response to lifestyle interventions [29], pharmacotherapy [20, 30–33], or psychotherapy [34].

To the best of our knowledge, only one meta-analysis has examined the association between substance use disorders and telomere length and has solely focused on cigarette smoking [35]. The results suggested a shorter telomere length among smokers compared with non-smokers with an inverse dose–response relationship with pack-years of smoking. Several empirical studies have been published on the relationship between telomere length and other SUDs, such as alcohol [36–38], cocaine [39], and other drugs [40, 41]. A recent non-systematic review has shown the inconsistency of the published results regarding the association between alcohol consumption and telomere length [42]. We are not aware of any systematic review or meta-analysis examining the association of telomere length with any other substance despite the extent and public health importance of substance use disorders.

The aims of the present study are (i) to systematically review the scientific literature on telomere length and SUDs to determine whether persons with SUDs have shorter telomere lengths compared with healthy controls, (ii) to explore potential differential effects with regard to diverse substances aside from nicotine, (iii) to identify potential moderators of the telomere length effect, and (iv) to investigate factors associated with potential heterogeneity in findings. To this end, the proposed systematic review will answer the following questions: (i) Do people with substance use disorders have shorter telomere lengths compared with healthy controls? (ii) Are there differences among substance users based on the type of substance that is misused? (iii) if heterogeneity is confirmed and substantive and methodological factors implicated in this heterogeneity. In particular, we will investigate the potential association of individual sociodemographic characteristics of the participants with the effect sizes. In addition, the potential confounding effects on the effect sizes will be investigated by analyzing the influence of methodological characteristics of the studies on the effect sizes, such as the design type and risk of bias items.

## Methods and analysis

The study protocol was reported according to the Preferred Reporting Items for Systematic Reviews and Meta-Analyses Protocol (PRISMA-P) 2015 statement [43] (see Additional file 1) and was registered prospectively (PROSPERO 2019 CRD42019119785, http://www.crd.york.ac.uk/PROSPERO).

### Searches

We will search PubMed/MEDLINE, EMBASE, PsycINFO, and Web of Science (from inception onwards) using the following search terms for SUDs: “drug, substance, addiction, alcohol*, heroin, cocaine, opium, opioid, methamphetamine, morphine” and for telomeres: telomeres, telomerase, and telo*” (see a draft search in Additional file 2). This search strategy was designed and will be carried out by a librarian expert (ARV). The reference lists of original studies included in this initial selection and review articles will then be manually searched to identify other potentially eligible studies. In addition, e-mails will be sent to the corresponding authors of selected studies in an attempt to identify potential unpublished studies that fulfill our selection criteria. To minimize potential publication bias, both published and unpublished papers will be eligible for inclusion and no restrictions are placed on time period, sample size, ethnicity, or language of publication.

### Eligibility Criteria

Studies will be selected according to the following criteria: (i) study design, (ii) participants, (iii) comparator, and (iv) outcome(s) of interest. (i) Study design: We will include observational studies (e.g., cohort, case–control, and cross-sectional studies) reporting telomere length in persons with SUDs compared with a control group (e.g., without SUDs). We will exclude studies in animals, experimental studies, reviews, case reports, case series, studies conducted in healthy populations, and/or families. (ii) Participants: We will include adults with SUD (regardless of age or sex) assessed by clinical interviews or established standard diagnostic instruments including, but not limited to, the Structured Clinical Interview for DSM-IV (SCID), Computerized National Institute of Mental Health Diagnostic Interview Schedule (CDISIV), Diagnostic and Statistical Manual of Mental Disorders (DSM-IV) criteria, or Composite International Diagnostic Interview (CIDI). We will exclude participants who exclusively meet the criteria for nicotine addiction. (iii) Comparator (or control group): The comparator group will be based on subjects with no history of SUDs (e.g., the general population, the community, unexposed outpatient, or hospital-based controls). (iv) Outcome(s): The outcome of interest will be telomere length.

### Selection and data collection

The results of the search strategy in the different databases will be combined in a unique file and duplicates and non-human research will be removed. Two investigators will independently review the title and abstract of each study identified through the search strategy to determine eligibility for inclusion. Full articles will be retrieved from the Internet or via library access when possible or by contacting corresponding authors when necessary. Data from each study will then be extracted independently using a previously defined protocol designed for data extraction and entered into two separate databases. In case of disagreement of study inclusion or in data extraction, a consensus will be reached with the involvement of the other researchers.

The following data will be extracted from each study: (i) author(s), journal, language, and year of publication; (ii) methods (study design, sample sizes for both cases and controls, diagnostic tools for the determination of case status, definition of case status and adjusted analyses, attrition for cases and controls and differential attrition); (iii) risk of bias assessment (described in greater detail below); (iv) sample characteristics for both cases and controls separately (gender ratio, mean age and standard deviation (SD), ethnic background, education level, type of substance use in cases, duration of substance use disorder in cases, presence of other mental disorders or medical illness, smoking status, exposure to childhood adversities and other stressful life events, and if the latest or any other variable has been measured before the onset of SUD in cases); and (v) telomere-related information (telomere length, tissue source, and telomere measurement method). The purpose of extracting all of these characteristics is to have the opportunity of examining their association with the effect sizes.

If additional groups of persons suffering from other psychiatric diagnoses are described in a study, only data from the substance use disorders sample and the control group will be extracted. The unit of analysis will be studies (rather than reports) to ensure data are not counted twice. As a result, in cases of multiple articles stemming from a single study, only the results of the publication with the highest number of participants will be included. If an article reports on two or more studies with independent samples, then each independent study will be included as an analysis unit in the meta-analysis. If essential data are missing from the study reports, the authors of the respective papers will be contacted and asked to provide additional data.

### Risk of bias (quality) assessment

The risk of bias of each study selected for inclusion will be assessed using the Newcastle–Ottawa Scale (NOS) for non-randomized studies in meta-analyses [44]. Studies will not be weighted by the overall quality score, and those with low values will not be excluded a priori. Discrepancies in the quality assessment of each study will be resolved by consensus. The influence on the effect size of each item assessed will be individually assessed [45].

### Strategy for data synthesis

#### Statistical analysis

For each study, the data will be converted into standardized mean differences (SMDs) as effect size index. The SMD is calculated as the mean difference in telomere length between the SUD (*M*_SUD_) and control (*M*_Control_) groups divided by the pooled standard deviation of the two groups (*S*): SMD = *c*(*m*)(*M*_SUD_ – *M*_Control_)/*S*, with *c*(*m*) being a correction factor for small sample sizes defined as *c*(*m*) = 1 − 3/(4 *N* – 9), *N* being the sum of the two sample sizes [46]. If means and standard deviations are not reported in the study, formulas will be applied to obtain the SMD from other statistical data (e.g., *t* tests, *F* tests, *p* values, correlations, odds ratios, etc.) [47]. Negative SMDs will represent a shorter telomere length for the SUD group compared with the control group. For each SMD, a 95% confidence interval (95% CI) will be calculated. In this meta-analysis, unadjusted effect sizes (SMDs) will be used. The potential influence of confounding factors will be assessed as described below.

Because a high level of heterogeneity is expected, random-effects models will be used a priori for SUD overall and for each independent substance consumed if the number of available studies allows it. Random-effects modeling assumes a genuine diversity in the results of the various studies and incorporates between-studies variance into the calculations. Random-effects models imply to weight each effect size by its inverse variance, this being the sum of the within-study and the between-studies variance. The between-studies variance will be estimated by restricted maximum likelihood [47]. Average effect size and a 95% CI will then be calculated with the improved method proposed by Hartung and Knapp [48–50]. Instead of assuming a standard normal distribution, the method by Hartung and Knapp assumes a Student *t* distribution with *k* – 1 degrees of freedom (*k* being the number of studies) and an improved estimator of the variance of SMD that takes into account the uncertainty in estimating the between-studies variance. In addition, a 95% prediction interval around the average effect size will be calculated, in order to provide a prediction of the expected true effects if a new study is conducted [47, 50].

To estimate heterogeneity between studies, the Cochran`s *Q* statistic, the *I*^2^ index, and visual inspection of the forest plots will be used. *Q* statistic is a weighted sum of the squared of the deviations of individual effect estimates from the overall estimate. A statistically significant result for the *Q* statistic is indicative of heterogeneity. The *I*^2^ index is calculated as *I*^2^ = 100(*Q* − *df*)/*Q*, with *df* being the degrees of freedom of the *Q* statistic: *df* = *k* – 1 (*k* being the number of studies). *I*^2^ is interpreted as the percentage of total variation across studies due to heterogeneity. The *I*^2^ index takes values between 0 and 100% with higher values denoting a greater degree of heterogeneity (0–25%: no or negligible heterogeneity; 25–50%: moderate heterogeneity; 50–75%: large heterogeneity; and 75–100%: extreme heterogeneity). *I*^2^ values of 25% or more will lead to investigate the influence of moderator variables. In addition, heterogeneity will be assessed with the between-studies variance and 95% confidence interval. Finally, following Mathur and VanderWeele’s (2019) proposal, the estimated proportion (and 95% confidence interval) of true effect sizes exceeding a scientifically meaningful threshold will be calculated. In terms of standardized mean difference, we will consider − 0.20 the threshold effect size for these calculations [51].

In cases of moderate-to-large heterogeneity (*I*^2^ > 25%), we will seek to identify possible explanations using subgroup analyses and meta-regressions based on the most important characteristics of the studies, including items used to evaluate the risk of bias. The analysis of moderating variables will be accomplished by assuming a mixed-effects model. Categorical moderators will be analyzed by comparing the average effect size of each category of the moderator [52], whereas continuous moderators will be analyzed by means of meta-regressions [53]. In both cases, the improved *F* statistic developed by Knapp and Hartung will be applied for testing the statistical significance of each moderator [54]. To estimate the proportion of variance accounted for by the moderator, an *R*^2^ index will be calculated [55].

Research on telomere length for persons with SUD must dedicate special attention to the potential influence of confounding factors. In order to address this point, several sensitivity analyses will be conducted. First, the risk of bias items of the NOS will be analyzed by means of subgroup analyses. Second, the comparability of the groups (cases and controls) is a key issue to assess the potential influence of confounding factors on effect sizes, with age being the most relevant confounding factor. In addition, to apply subgroup analyses to the risk of bias item on comparability of the NOS, simple meta-regressions will also be conducted using the SMDs as a dependent variable and the mean difference in age between cases and controls, the difference in age SDs, the difference in the proportion of males and of Caucasians, and the difference in education levels. Third, another set of subgroup analyses will be conducted with other covariates related to telomere length, such as smoking status, exposure to childhood adversities or other stressful events, and the presence of mental or physical comorbidities.

The presence of publication bias will be examined using the “funnel plot” method using Duval and Tweedie’s trim-and-fill method [56], the Egger test [57], and the precision-effect test–precision-effect estimate with standard error (PET-PEESE) method [58]. A sensitivity analysis will then be performed to assess whether our results were substantially influenced by the presence of any individual study by systematically removing each study and recalculating the significance of the overall results. If conditions impede meta-analysis, data will be narratively presented. All statistical analyses will be conducted using the metafor program in R [59]. To judge the quality of evidence for all outcomes, the Grading of Recommendations Assessment, Development and Evaluation (GRADE) approach will be used [60]. Results will be reported according to the Preferred Reporting Items for Systematic Reviews and Meta-Analyses (PRISMA) statement [61].

## Discussion

To the best of our knowledge, this systematic review and meta-analysis will be the first to systematically assess the association of telomere length with any other substance despite the extent and public health importance of substance use disorders. The results of this systematic review should be interpreted with caution as there are some potential limitations at the study and review level. At the study level, (i) the study design of the original studies might limit causal relation as cross-sectional and case–control designs are expected to be found, rather than longitudinal studies, and (ii) a high level of heterogeneity in the quality of the designs is expected. At the review level, (i) the analyses of this meta-analysis will be based on unadjusted estimates as different adjustments by different potential confounders are expected in original studies. The potential impact of those moderating variables (age, gender, tobacco smoking, among others) will be assessed by a combination of subgroup and meta-regression analyses; (ii) differences in the tissue types and methods used to measure telomere length is expected; and (iii) the scarcity of studies may limit some subgroup or stratified analyses individual substances.

This review will provide information on potential moderators of the telomere length effect related to an expected heterogeneity in published scientific literature. The assessment of risk of bias (or “quality”) of individual studies included and how the results might influence the review findings will be useful for researchers in future projects.

No ethical issues are anticipated. The results of this systematic review and meta-analysis will be published in a peer-reviewed journal or conference presentation. Should any amendment be introduced in the present protocol, a description of the changes and their rationale with the date of incorporation will be updated in the PROSPERO register and be acknowledged in the publication of the results.

## Supplementary information

**Additional file 1.** Preferred Reporting Items for Systematic Review and Meta-Analysis Protocols (PRISMA-P) 2015 Checklist: recommended items to include in a systematic review protocol.

**Additional file 2.** Search strategy by electronic database: MEDLINE, EMBASE, PsycINFO and Web of Science (WOS).

## Data Availability

All data generated and datasets use during the current study will be available from the corresponding author on reasonable request.

## Abbreviations

CDISIV: Computerized National Institute of Mental Health Diagnostic Interview Schedule
CIDI: Composite International Diagnostic Interview
DNA: Deoxyribonucleic acid
DSM-IV: Diagnostic and Statistical Manual of Mental Disorders criteria
GRADE: Grading of Recommendations Assessment, Development and Evaluation
NOS: Newcastle–Ottawa Scale
PRISMA: Preferred Reporting Items for Systematic Reviews and Meta-Analyses
PRISMA-P: Preferred Reporting Items for Systematic reviews and Meta-Analyses Protocols
PROSPERO: International Prospective Register of Systematic Reviews
SCID: Structured Clinical Interview for DSM-IV
SD: Standard deviation
SMD: Standardized mean differences
SUD: Substance use disorders
