# Supplementary material for: The association of telomere length with substance use disorders: systematic review and meta-analysis protocol
Source: Syst Rev. 2019 Dec 1;8:298. doi: 10.1186/s13643-019-1199-x (PMC6886210; doi:10.1186/s13643-019-1199-x)

Additional file 2: Search Strategy

**PUBMED**
(("drug abuse" OR "drug addiction" OR "drug dependence" OR "drug use disorder" OR "drug habituation" OR "substance abuse" OR "substance addiction" OR "substance dependence" OR (drug*[tiab] AND abuse[tiab]) OR (drug*[tiab] AND addiction[tiab]) OR (drug*[tiab] AND dependence[tiab]) OR (drug*[tiab] AND habituation[tiab]) OR (substance[tiab] AND abuse[tiab]) OR (substance[tiab] AND addiction[tiab]) OR (substance[tiab] AND dependence[tiab]) OR "Substance-Related Disorders"[Mesh] OR alcohol* OR heroin OR cocaine OR cannabi* OR marijuana OR opium OR *amphetamine OR amphetamine* OR morphine OR LSD OR "Lysergic Acid Diethylamide" OR opio* OR "street drugs"[MeSH] OR "Psychotropic Drugs"[MeSH] OR "Designer Drugs"[MeSH]))<br>AND<br>("Telomere Shortening"[Mesh] OR "telomere length" OR "Telomere Shortening"[tiab] OR "Telomere"[Mesh])
**PsychINFO-Psychlit**
(DE "Drug Abuse" OR DE "Drug Addiction" OR DE "Drug Dependency" OR DE "Substance Abuse and Addiction Measures" OR DE "Substance Use Disorder" OR "drug abuse" OR "drug addiction" OR "drug dependence" OR "drug use disorder" OR "drug habituation"<br>OR "substance abuse" OR "substance addiction" OR "substance dependence"<br>OR ((TI drug* OR TI substance*) AND (TI abuse OR TI addiction OR TI dependenc* OR TI habituation))<br>OR ((AB drug* OR AB substance*) AND (AB abuse OR AB addiction OR AB dependenc* OR AB habituation))<br>OR MA "Substance-Related Disorders" OR MA "street drugs" OR "street drugs"[MeSH] OR MA "Psychotropic Drugs" OR "Psychotropic Drugs"[MeSH] OR MA "Designer Drugs" OR "Designer Drugs"[MeSH] OR alcohol* OR heroin OR cocaine OR cannabi* OR marijuana<br>OR opium OR *amphetamine OR methamphetamine* OR amphetamine* OR morphine OR opio* OR "Lysergic Acid Diethylamide" OR "LSD")<br>AND<br>telomer*
**EMBASE**
('drug abuse' OR 'drug addiction' OR 'drug dependence'/exp OR 'drug use disorder' OR 'drug habituation' OR 'substance abuse' OR 'substance addiction' OR 'substance

dependence' OR (drug*:ti,ab AND abuse:ti,ab) OR (drug*:ti,ab AND addiction:ti,ab) OR (drug*:ti,ab AND dependenc*:ti,ab) OR (drug*:ti,ab AND habituation:ti,ab) OR (substance:ti,ab AND abuse:ti,ab) OR (substance:ti,ab AND addiction:ti,ab) OR (substance:ti,ab AND dependenc*:ti,ab) OR alcohol* OR heroin OR cocaine OR cannabi* OR marijuana OR opium OR methamphetamine* OR amphetamine* OR morphine OR opio* OR LSD OR 'Lysergic Acid Diethylamide' OR 'street drug'/exp OR 'designer drug'/exp OR 'illicit drug'/exp OR 'recreational drug'/exp)<br>AND<br>('telomere shortening'/exp OR 'telomere length'/exp OR 'telomere shortening':ti,ab OR 'telomere length':ti,ab OR 'telomere'/exp)
**WOS**
(((TS=drug* OR TS=substance*) AND (TS=abuse OR TS=addiction OR TS=dependenc* OR TS=habituation)) OR TS="street drugs" OR TS="Psychotropic Drugs" OR TS="Designer Drugs" OR TS=alcohol* OR TS=heroin OR TS=cocaine OR TS=cannabi* OR TS=marijuana OR TS=opium OR TS=methamphetamine* OR TS=amphetamine* OR TS=morphine OR TS=opio* OR TS=LSD OR TS ="Lysergic Acid Diethylamide")<br>AND<br>TS=Telomere*

FLOW CHART OF THE PROCESS FOR SEARCHING AND ASSESSING ELIGIBILITY OF STUDIES (Adapted from Sagoo y cols, PLOSMedicine 2009; 6 (3): e1000028)

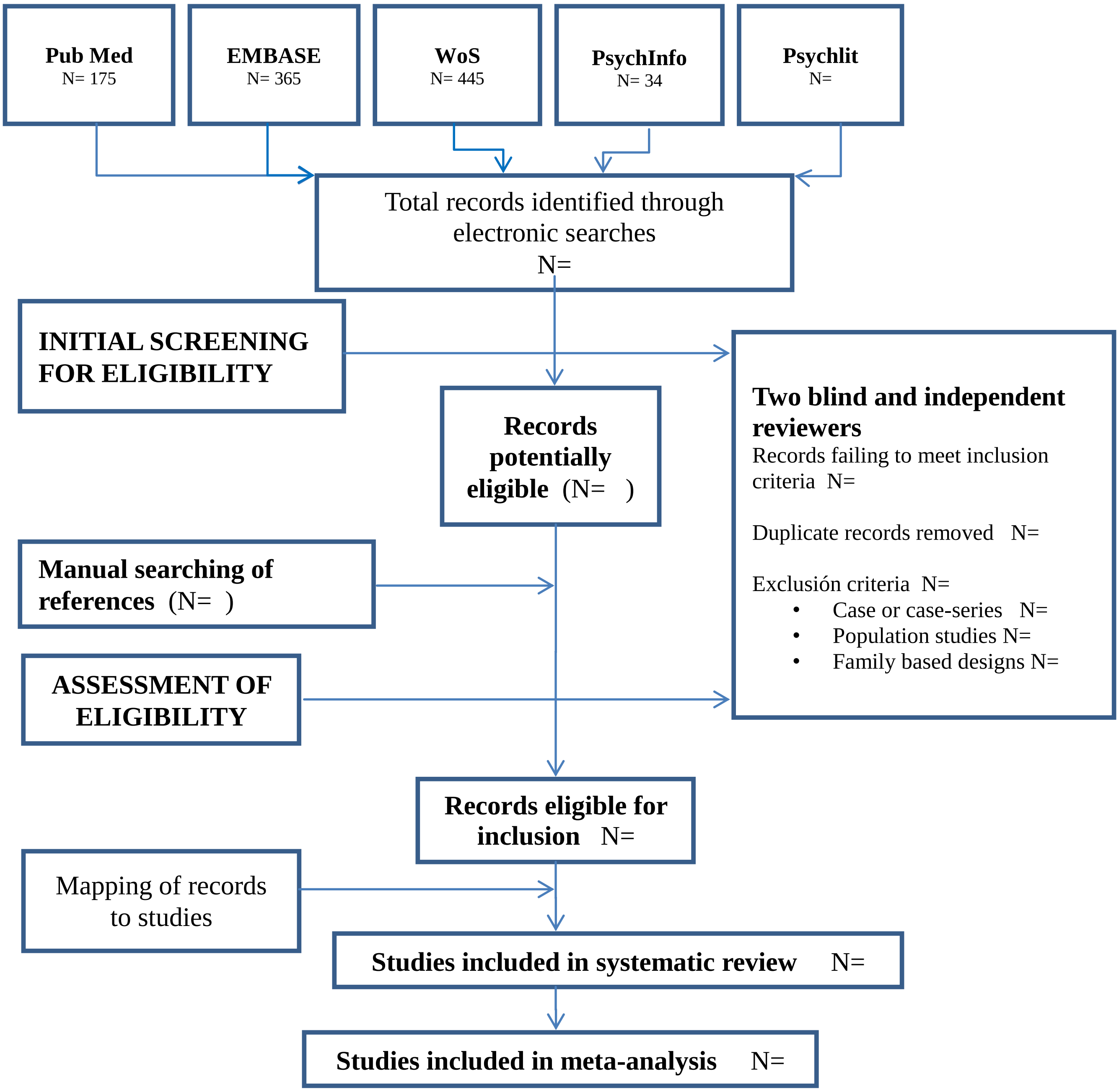

Supplement: Supplementary file 2 — Additional file 2. Search strategy by electronic database: MEDLINE, EMBASE, PsycINFO and Web of Science (WOS). [file 13643_2019_1199_MOESM2_ESM.pdf]
